# The Determination of Blood Glucose Lowering and Metabolic Effects of *Mespilus germanica* L. Hydroacetonic Extract on Streptozocin-Induced Diabetic Balb/c Mice

**DOI:** 10.3390/medicines5010001

**Published:** 2018-01-01

**Authors:** Fatemeh Shafiee, Elnaz Khoshvishkaie, Ali Davoodi, Ayat Dashti Kalantar, Hossein Bakhshi Jouybari, Ramin Ataee

**Affiliations:** 1 Student Research Committee, Pharmaceutical Sciences Research Center, Mazandaran University of Medical Sciences, Sari 4847193698, Iran; fatemehshafiee0@gmail.com (F.S.); Elnaz.K@yahoo.com (E.K.); 2 Department of Pharmacognosy, Faculty of Pharmacy, Mazandaran University of Medical Sciences, Sari 4847193698, Iran; adavoodi.pharm@gmail.com (A.D.); ma_2253@yahoo.com (H.B.J.); 3 Medicinal Plants Research Center, Ayatollah Amoli Branch, Islamic Azad University, Amol 4635143358, Iran; adavoodi.pharm@gmail.com; 4 Pharmaceutical Sciences Research Center, Hemoglobinopathy Institute, Mazandaran University of Medical Sciences, Sari 4847193698, Iran; dashti68612@gmail.com (A.D); raminataee1349@gmail.com (R.A); 5 Thalassamia Research Center, Hemoglobinopathy Institute, Mazandaran University of Medical Sciences, Sari 4847193698, Iran; raminataee1349@gmail.com

**Keywords:** flavonoids, diabetes, Rosaceae, *Mespilus germanica*, mice

## Abstract

**Background:** The serum glucose lowering, normalization animal body weight, and antioxidative stress effects of *Mespilus germanica* L. leaf extract were investigated in normal and streptozotocin-induced Balb/C mice. **Methods:** The phenol and flavonoid of the leaves of *M. germanica* were extracted by percolation and concentrated using a rotary evaporator. Its total phenol and flavonoid content was determined using folin and aluminum chloride methods, respectively. The study was conducted on 48 matured male Balb/C mice (20–30 g) divided into 6 groups (*n* = 8). Diabetes mellitus was induced by single intraperitoneal injection of 35 mg/kg of streptozotocin (STZ). Extracts of *Mespilus germanica* were used orally at the dose of 50, 100, and 200 mg/kg body weight per day for 21 days. **Results:** Oral administrations of the *M. germanica* L. leaf extract significantly decreased serum glucose, oxidative stress, and lipid peroxidation and maintained animal body weight during treatment period (*p* < 0.05) compared to metformin (200 mg/kg) in over 100 mg/kg, 200 mg/kg, and 50 mg/kg dosages, respectively. **Conclusions:** The present study indicated that the *Mespilus germanica* leaf extract significantly decreased serum glucose and maintained normal body weight in Balb/C diabetic mice.

## 1. Introduction

Diabetes mellitus (DM) is a metabolism disorder and exocrine system dysfunction that represent an insulin deficiency or resistance of cells for insulin hormone. DM can be classified in two basic types, Type I and Type II, Type I DM resulting from the pancreatic cell’s failure to produce endogen insulin. Type II DM illustrates a condition of quickly hyperglycemia due to cell insulin receptors resistance [1,2]. The present number (as of 2014) of diabetic humans in worldwide is about 300 million, and this is likely to be increased to about 600 million or more by the year 2030. Reasons for this include a decrease in lifestyle levels, the consumption of high energetic diets, and obesity. [3].

Many medicinal plants have been recommended for the treatment of diabetes mellitus. Drugs of plant sources are frequently considered to be less toxic and almost free from adverse effects in conventional uses [4,5,6]. *Mespilus germanica* is a large shrub or small tree common in northern forest regions of Iran that grows to a height of 2–6 m. It is a member of the Rosaceae family and has very nutritive and therapeutic usages in Iran. The fresh and dried fruits and leaves of the plant are usually used in treating wounds, oral abscess, diabetes mellitus, microbial infections, etc. [7,8,9]. *M. germanica* fruits are rich in phytochemicals, nutrition, and therapeutic ingredients. It contains proteins, carbohydrates, lipids, and phenolic compounds such as flavonoids and tannins. These phytochemicals induce the therapeutic effects of the plant [10].

Despite the absence of studies on the anti-diabetic effect of this plant, except for those proposed for the measurement of its phenol and flavanoid components and the anti-oxidative stress properties of the related compounds, this research was designed to experimentally determine the serum glucose lowering, normalization animal body weight, and antioxidative stress effects of *Mespilus germanica* leaf hydro acetonic extract used in normal and streptozotocin-induced Balb/c mice.

## 2. Materials and Methods

### 2.1. Plant

*Mespilus germanica* leaves were collected from Jouybar city of Mazandaran province in Iran (latitude: 36.644272; longitude: 54.963289) in the spring of 2015. The plant was identified by Department of Pharmacognosy, and Faculty of Pharmacy of Mazandaran University of Medical Sciences in Sari, Iran. The voucher number of this plant is defined as E_1_-223202. The voucher specimen is stored in the Department of Pharmacognosy Herbarium.

### 2.2. Extract Preparation

Fresh *M. germanica* leaves were dried and placed in a percolator to extract with 70% acetone via percolation. Briefly, 100 g of powdered leaves were macerated in 1000 mL solvents for 24 h. Then, the same amount of solvent was used for continuous extraction. After extraction, the solvent was evaporated in 40 °C with a rotavap, and extracts were freeze-dried. The obtained acetonic extract was stored at −10 °C until being used [11].

### 2.3. Phenols and Flavonoids Assay

Total phenolic contents of *M. germanica* leaf extract were determined by Folin–Ciocalteu’s method. The Folin reagent was diluted 2-fold with distilled water. One milliliter of extracts (1 mg/mL) was added to 1.5 mL of reagent and allowed to stand at room temperature for 5 min. Sodium carbonate solution (1.25 mL, 20%) was added to the mixture and stored at room temperature for an additional 60 min, and absorptions at 725 nm were recorded. Calibration curve was created by a standard concentration of tannic acid and total phenolic compounds of extract were obtained by calibration curve [12,13].

The total flavonoid content of the extract was determined by aluminum chloride (ALCl_3_). The sample solution (1 mL) was mixed with 1 mL of aluminum trichloride (2%) in methanol. Moreover, a blank solution was prepared by adding an extract solution (1 mL) to 1 mL of methanol without AlCl_3_. The extract and blank absorbance were recorded at 415 nm after 10 min of incubation at 25 °C. The total flavonoid content was expressed as equivalents of quercetin. Furthermore, calibration curve was prepared by a standard solution of quercetin [12,14].

### 2.4. Animal Studies

#### 2.4.1. Animal Conditions

Male Balb/C mice weighing 20–30 g were housed in clean cages at room temperature (22–25 °C), 12-h light/12-h dark cycle and relative air humidity 40–60%. Mice had continuous access to food and tap water. All procedures involving animals were approved by the ethical committee of Mazandaran University of Medical Sciences. With ethical code 294 , 25 August 2016.

#### 2.4.2. Preparation of Diabetic Mice

The animals were injected with streptozocin (35 mg/kg, IP). Five days after injection, the mice with fasting blood glucose higher than 250 mg/dL were used for the experiments. Eight mice were used in each experiment. In addition, each animal was used once only in all of experiments. The dietary food and water were removed from cages 12 h before testing [5].

#### 2.4.3. Drug Administration

The extract was suspended in distilled water and administered orally through oro-gastric tube at doses of 50, 100, and 200 mg/kg body weight. The volume of administrated extract was calculated 1 unit of insulin syringe per gram of weight for each animal [5].

#### 2.4.4. Experimental Design

In the present study, 48 Balb/C mice (40 diabetics, 8 normal mice) were used. The mice were divided into six groups. In addition, eight mice were used in each group.

*Group 1*: Normal control mice were administrated 1 unit of insulin syringe per gram of weight of distilled water daily for 21 days.

*Groups 2*: Diabetic mice were administrated 1 unit of insulin syringe per gram of weight of distilled water daily for 21 days.

*Group 3*: Diabetic mice were administrated metformin (200 mg/kg body weight) daily for 21 days.

*Groups 4–6*: Diabetic mice were administrated *M. germanica* extract (50, 100, and 200 mg/kg body weight) daily using a gavage tube for 21 days [5].

#### 2.4.5. Serum Glucose Assay

After 21 days of treatments, blood samples were drawn from the hearts of the mice, and serum glucose was determined. Serum glucose was estimated with a digital blood glucose analyzer [13].

#### 2.4.6. Glutathione and Lipid Peroxidation Assay

##### Tissue Preparation and Subcellular Fractionation

The animal groups were killed after 21 days, and livers were taken out and kept on a low temperature condition. The livers were homogenized separately in a phosphate buffer (10 mM phosphate buffer, pH 7.0) and centrifuged at 1000× *g* for 10 min at 4 °C to separate the nuclear debris. The aliquot obtained was used for the lipid peroxidation [15].

##### Lipid Peroxidation Assay

The procedure was used for the estimation of the rate of lipid peroxidation. Homogenate tissue (0.5 mL) was pipetted in a 15 × 100 mm glass tube and incubated at 37 ± 1 °C in a shaker (100 cycles per min) for 60 min. Another 0.5 mL of the same homogenate was pipetted in a centrifuge tube and placed at 0 °C. After 1 h of incubation, 0.5 mL of 5% chilled TCA followed by 1 mL of 0.67% TBA was added to each glass tube and centrifuge tube and mixed after each addition. The aliquot amounts of each tube were centrifuged at 2000× *g* for 20 min. Then, the supernatant was placed in a boiling water bath. After 10 min, the test tubes were taken out and cooled, and the absorbance of the color was read at 535 nm [15].

##### Glutathione Assay

Reduced glutathione was assayed using a spectroscopic method. Post-mitochondrial supernatant (0.5 mL) was precipitated with 0.5 mL of sulfosalicylic acid (2%). The samples were kept at 4 °C for 2 h and then subjected to centrifugation at 1500× *g* for 10 min at 4 °C. The assay mixture contained 0.5 mL of filtered residue, 2.3 mL of phosphate buffer (0.2 M, pH 7.4), and 0.2 mL of DTNB (0.5% in phosphate buffer 0.2 M, pH 7.4) and was calculated immediately at 412 nm [15,16].

#### 2.4.7. Statistical Analysis

Statistical analyses were performed using one-way analysis of variance and a Student’s *t*-test by SPSS 16. The differences between the means were considered significant at the probability level *p <*0.05 [17].

## 3. Results

**Figure 1:**
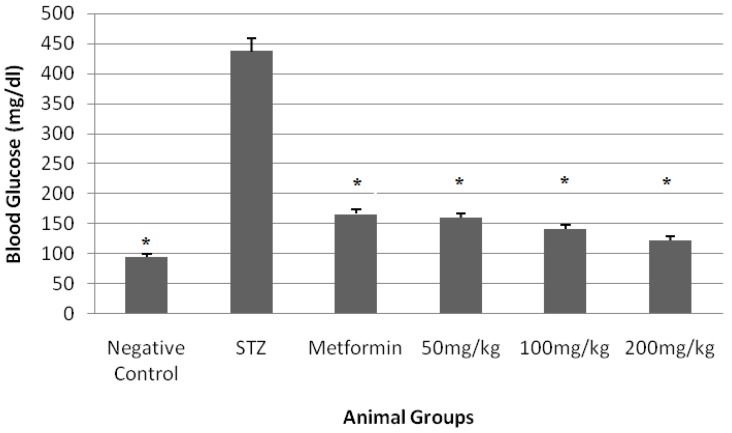
Effect of extract on blood glucose (mg/dL) in mice. * *p* < 0.05 compared with Streptozocin (STZ).

**Figure 2:**
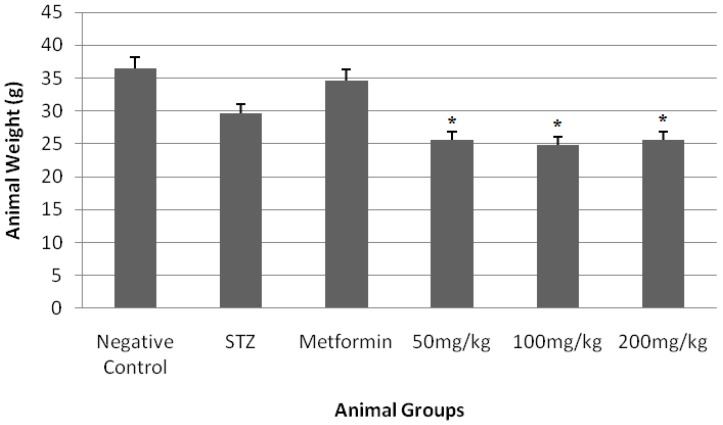
Effect of extract on body weight in mice (g). * *p* < 0.05 compared with negative control.

The dried hydroacetonic extracts yielded 18.6% (*w*/*w*). The total phenol and flavonoid content of the extract were 720 and 500 (mg/100 g), respectively. The blood sugar levels and animal weights measured in normal and experimental Balb/C mice before and after 21 days of treatment are shown in Figure 1 and Figure 2.

**Figure 3:**
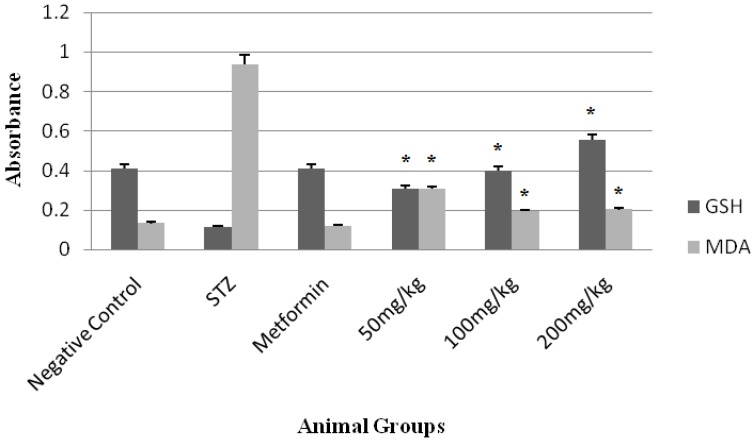
Effects of extract on stress oxidative and lipid peroxidation in mice. GSH: glutathione as an indicator of oxidative stress; MDA: malonyl dealdehyde as an indicator for lipid peroxidation; * *p* < 0.05 compared with STZ.

Additionally, the preventive effects of *M. germanica* leaf extract on stress oxidative and lipid peroxidation in Balb/C mice shown in Figure 3.

According to the results shown in Figure 1, the extract reduced the blood sugar almost in a dose-dependent manner, more evenly than metformin at doses of 100 and 200 mg/kg; however, as shown in Figure 2, the extract reduced the weight unexpectedly but was maintained in a constant range.

As in Figure 3, STZ had reduced glutathione (GSH) as an indicator for oxidative stress and increased malonyl dealdehyde as an indicator for lipid peroxidation. Metformin prevented MDA reducing and GSH increasing and the extract reduced MDA and increased GSH in a dose-dependent manner.

## 4. Discussion

According to the obtained data, *M. germanica* leaf hydroacetonic extract is effective in reducing blood glucose. This extract also significantly increased glutathione and decreased lipid peroxidation in a dose-dependent manner. Although the extract could not improve the weight of animal and evenly decreased the weights, this reduction was maintained, was not dose-dependent, and may be due to toxicities of the extract. As the extract had anti-oxidative stress properties, its sugar-reducing effect was most likely due to its protection effect on mitochondrias of the liver and not because of its toxicities. Additionally, the high total phenol and flavonoid contents of the extract can emphasize its anti-oxidant effects and the decreasing intestinal absorption of dietary sugars, which can also explain its weight reductions.

In diabetic conditions, auto-oxidation of excessive glucose leads to an accumulation of reactive oxygen species (ROS), the overproduction of which is said to be related to complications associated with diabetes. This has been supported by studies reporting improvement in diabetic complications through antioxidant treatment, including antioxidant chemicals or plant extracts [18]. Indeed, the underlying DM induction mechanism of STZ is based on the formation of superoxide radicals in pancreatic beta cells in consequence of several reactions [18]. GSH is considered a non-enzymatic antioxidant and has free radical scavenging potential in the cellular defense system [18]. MDA is an end product of lipid peroxidation and is a marker of oxidative stress related to membrane damage [18]. *M. germanica* extracts are comprised of polyphenolic compounds that are known to have antioxidant properties. One of these compounds, quercetin, is a flavonoid and is also able to inhibit glucose absorption in the intestines [18,19,20,21].

In our study, elevated MDA levels and decreased GSH activities in diabetic rats proved the key role of oxidative stress in the pathophysiology of liver damage in DM, and the extract could improve them.

Different studies have shown antidiabetic effects for some plants due to the presence of phytochemicals in their herbal organs [19,20]. Flavonoids and other phenolic compounds are potentially antidiabetic effects by glucose absorption inhibition in gastro-intestinal tract [21] and the antidiabetic effect of *M. germanica* is most likely due to flavanoid content such as quercetin.

Our study was also in parallel with other studies, such as those by Panjeshahin MR et al. on the antidiabetic activity of different extracts of *Myrtus communis* in streptozotocin-induced diabetic rats [5], those by Sokri G et al. on the anti-diabetic effects of the hydroalcoholic extract of green tea and cinnamon on diabetic rats [13], and those by Hatice B et al. on the anti-diabetic effects of aqueous extract of *Myrtus communis* L. leaves in diabetic rats [18]. In all of these studies, the herbal extract role of phenol and flavanoids such as quercetin as anti-oxidative stress compounds have been considered. Since we found a high percentage of phenols and flavanaoids in *M. germanica*, we can suggest that the compounds of this plant have an anti-diabetic effect, but more studies are needed for purifications of these components and for more precise toxicology and pharmacologic assay.

## 5. Conclusions

The present study indicated that the *Mespilus germanica* leaf extract significantly decreased serum glucose and maintained normal body weight in Balb/C diabetic mice as compared with control groups. In addition, this extract decreased oxidative stress and lipid peroxidation. In conclusion, this species and other citable plants are very valuable and should be evaluated in experimental and clinical trials for their pharmacological efficacy and the discovery of new approved drugs for diabetes mellitus.
